# The Molecular Mechanisms Governing the Assembly of the Immuno- and Thymoproteasomes in the Presence of Constitutive Proteasomes

**DOI:** 10.3390/cells11091580

**Published:** 2022-05-07

**Authors:** Ayaka Watanabe, Hideki Yashiroda, Satoshi Ishihara, Megan Lo, Shigeo Murata

**Affiliations:** Laboratory of Protein Metabolism, Graduate School of Pharmaceutical Sciences, The University of Tokyo, 7-3-1 Hongo, Bunkyo-ku, Tokyo 1130033, Japan; watanabe-ayaka194@g.ecc.u-tokyo.ac.jp (A.W.); yashiroda@mol.f.u-tokyo.ac.jp (H.Y.); ishihara-1-2-3@g.ecc.u-tokyo.ac.jp (S.I.); mlo@g.ecc.u-tokyo.ac.jp (M.L.)

**Keywords:** proteasome, immunoproteasome, thymoproteasome, intermediate proteasome, chaperone, propeptide, PAC1–PAC2, PAC3–PAC4, UMP1

## Abstract

The proteasome is a large protein complex responsible for proteolysis in cells. Though the proteasome is widely conserved in all eukaryotes, vertebrates additionally possess tissue-specific proteasomes, termed immunoproteasomes and thymoproteasomes. These specialized proteasomes diverge from constitutive proteasomes in the makeup of their catalytic 20S core particle (CP), whereby the constitutive β1, β2, and β5 catalytic subunits are replaced by β1i, β2i, and β5i in immunoproteasomes, or β1i, β2i, and β5t in thymoproteasomes. However, as constitutive β1, β2, and β5 are also present in tissues and cells expressing immuno- and thymoproteasomes, the specialized proteasomes must be able to selectively incorporate their specific subunits. Here, we review the mechanisms governing the assembly of constitutive and specialized proteasomes elucidated thus far. Studies have revealed that β1i and β2i are added onto the α-ring of the CP prior to the other β subunits. Furthermore, β5i and β5t can be incorporated independent of β4, whereas constitutive β5 incorporation is dependent on β4. These mechanisms allow the immuno- and thymoproteasomes to integrate tissue-specific β-subunits without contamination from constitutive β1, β2, and β5. We end the review with a brief discussion on the diseases caused by mutations to the immunoproteasome and the proteins involved with its assembly.

## 1. Introduction

**Figure 1:**
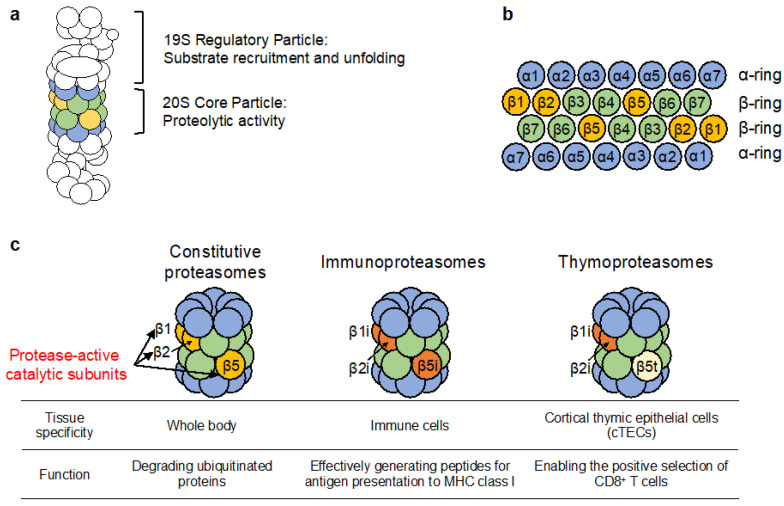
Schematic diagram of the proteasome. (**a**) The 26S proteasome consists of the catalytic 20S core particle and the 19S regulatory particle. (**b**) The subunits composing the 20S core particle. The catalytic β1, β2, and β5 subunits are responsible for caspase-like, trypsin-like, and chymotrypsin-like activities, respectively. (**c**) Tissue specific proteasomes are generated by switching their catalytic subunits.

The 26S proteasome is a widely conserved protein complex that is responsible for the recognition and selective intracellular proteolysis of ubiquitinated proteins in eukaryotes [1]. The large, 4.3 MDa proteasome complex consists of 33 distinct subunits that together comprise the 20S core particle (CP), which possesses proteolytic activity, and one or two 19S regulatory particles (RP), which are responsible for regulatory functions such as the recruitment and unfolding of substrate proteins. The CP is formed by the axial stacking of four heteroheptameric rings: two outer α-rings and two inner β-rings, each made up of seven structurally similar yet distinct α and β subunits, creating a α_1–7_β_1–7_β_1–7_α_1–7_ structure (Figure 1). Of these 14 subunits, the β1, β2, and β5 subunits are responsible for caspase-like, trypsin-like, and chymotrypsin-like activities, respectively.

Though the CP of the constitutive proteasome (cCP) is conserved as far back as unicellular eukaryotes, cartilaginous fish and higher-order vertebrates additionally express a specialized proteasome, the immunoproteasome, in which the constitutive catalytic subunits β1, β2, and β5 are replaced by the immune subunits β1i/LMP2, β2i/MECL1, and β5i/LMP7 [2]. The expression of the immunoproteasome is tissue-specific and is highest in immune cells and IFN-γ-stimulated cells. The replacement of the constitutive catalytic subunits for the immune subunits of the immunoproteasome results in reduced caspase-like activity, but enhanced chymotrypsin-like and trypsin-like activities compared to that of the cCP. This change in activity allows the immunoproteasome to produce a greater number of peptides with hydrophobic or basic C-termini than the cCP. As a result, the immunoproteasome is thought to contribute to the maintenance of the immune system function by producing peptides suitable for antigen presentation to MHC class I. Indeed, knockout of any one of the specialized immune subunits leads to alterations to MHC class I expression or T cell function [3,4,5]: β1i knockout mice display a reduced number of CD8^+^ T lymphocytes despite having no changes to the expression levels of MHC class I [5]. Furthermore, β2i-deficient mice possess an altered T cell repertoire [3]. Lastly, β5i knockout mice also exhibit a 50% reduction in MHC class I expression and impaired antigen presentation to cytotoxic T-cells [4].

While much has been elucidated about the critical role the immunoproteasome plays in the maintenance of MHC class I and T cell function, less is understood about the second type of specialized proteasomes possessed by vertebrates. Cortical thymic epithelial cells (cTECs) express a specific proteasome termed the thymoproteasome, which is required for the differentiation of CD8^+^ T cells [6]. Similar to the immunoproteasome, the thymoproteasome contains the specialized β1i and β2i subunits. However, the thymoproteasome diverges from the immunoproteasome in its β5t catalytic subunit, which is specifically expressed in cTECs. Though the substrate preference of β5t remains unclear, the thymoproteasome was shown to possess less chymotrypsin activity than the immunoproteasome, which incorporates β5i instead of β5t. An important step in CD8^+^ T cell differentiation is their interaction with MHC class I expressed on the surface of cTECs with moderate affinity. Therefore, the thymoproteasome may contribute to CD8^+^ T cell differentiation by using its specific protein cleavage activity to produce specialized MHC class I-binding peptides in cTECs.

In contrast to the catalytic subunits of the immuno- and thymoproteasome, whose expressions are constricted to specialized tissues and cells, the constitutive catalytic subunits, β1, β2, and β5 of the cCP are expressed ubiquitously. That is to say, the tissues and cells in which the immuno- and thymoproteasome are expressed also contain the constitutive catalytic subunits. It is therefore imperative for the cCP, the immuno-, and the thymoproteasome to be able to selectively incorporate their specific set of subunits. In this review, we outline the different mechanisms of cCP, immuno-, and thymoproteasome assembly, and how each proteasome incorporates the correct set of β-subunits in the presence of a mixture of different β-subunits.

## 2. Assembly of the cCP

**Figure 2:**
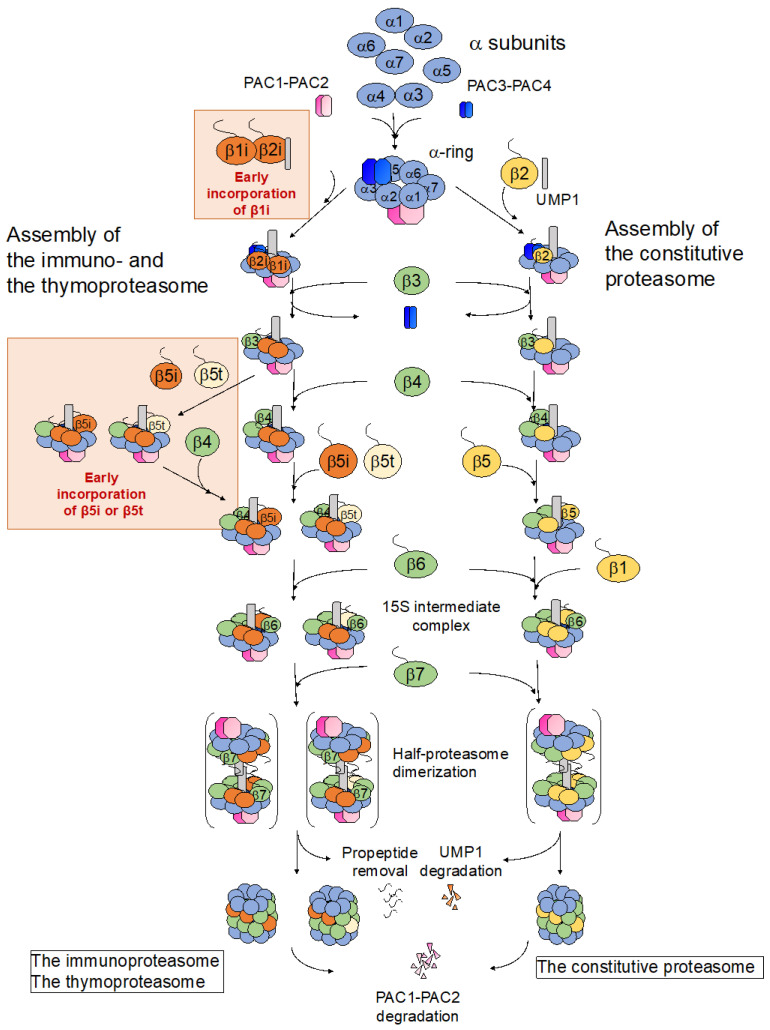
Assembly pathways of the constitutive, the immuno-, and the thymoproteasomes. There are two differences between the assembly pathway of the constitutive proteasome and that of the immuno- and the thymoproteasomes. The first difference is that β1i is incorporated earlier than constitutive β1. The second is that constitutive β5 is incorporated after β4, whereas β5i and β5t can be incorporated on the α-ring independent of β4, and either β4 or β5i and β5t can be incorporated into the β-ring immediately after the incorporation of β3.

The assembly of the cCP begins with the formation of the α-ring, followed by binding of the β subunits to the α-ring to form a half-proteasome consisting of one α-ring and one β-ring. The fully assembled cCP is completed upon the association of two half-proteasomes (Figure 2) [1,7,8,9].

### 2.1. Assembly of the α-Ring Is Aided by Highly Conserved Chaperone Complexes

The archaeon *Thermoplasma acidophilum* expresses a single type of α and β subunit, which forms its 20S CP. When expressed alone in *E. coli*, this α subunit is able to form a heptameric α-ring. However, in contrast, the β subunit when expressed alone, is unable to form a heptameric β-ring without the presence of α subunits [10,11]. Thus, the assembly of the cCP is assumed to begin with the formation of the α-ring. Eukaryotes possess seven homologous yet distinct α subunits (α1−7), and the proper formation of the α-ring from these seven subunits is dependent on the Proteasome Assembly Chaperone-1 (PAC1)–PAC2 complex (the budding yeast ortholog is Pba1–Pba2) and the PAC3–PAC4 complex (the budding yeast ortholog is Pba3–Pba4) [12,13,14,15,16,17,18,19]. 

The PAC1–PAC2 complex was originally identified as a proteasome-binding complex in human cells [13]; mammalian cells lacking PAC1–PAC2 have reduced expression of the complete α-ring, and instead form off-pathway products that appear to be dimeric α-rings. Similarly, in budding yeast, Δ*pba1* cells form unstable α-rings, where α5 and α6 can be easily dissociated [20]. All eukaryotic PAC1/Pba1 homologues possess a conserved HbYX motif (Hb is a hydrophobic amino acid, Y is tyrosine, and X is any amino acid) at their C-termini. In budding yeast, the C-terminus of Pba2 also contains an HbYX motif, and the HbYX motifs of Pba1 and Pba2 are required for their ability to bind to the α5−α6 and α6−α7 pockets, respectively [21,22]. The HbYX motif also exists in the Rpt2, 3, and 5 subunits of the RP, which allows them to bind to the α-ring and open the CP. Thus, Pba1–Pba2 and the RP bind competitively to the outside of the α-ring [23,24]. However, Pba1–Pba2 has a higher affinity than the RP for immature CPs, whereas the RP has a higher affinity than Pba1–Pba2 for mature CPs [20]. As a result, the Pba1–Pba2 complex suppresses the association of the RP with immature CPs and assists with the proper assembly of the α-ring. As the α1–4 subunits of the CP all contain nuclear localization signals, the association of PAC1–PAC2 with immature CPs also functions to retain CP intermediates in the cytoplasm so that they are not transported to the nucleus prematurely [25]. Instead, the 26S proteasome is first fully assembled in the cytoplasm before being transported into the nucleus. Intermediates of the α-ring formed upon knockdown of α1 fractionate into cytoplasmic fractions, whereas those formed under knockdown of both α1 and PAC1 fractionate into nuclear fractions.

**Figure 3:**
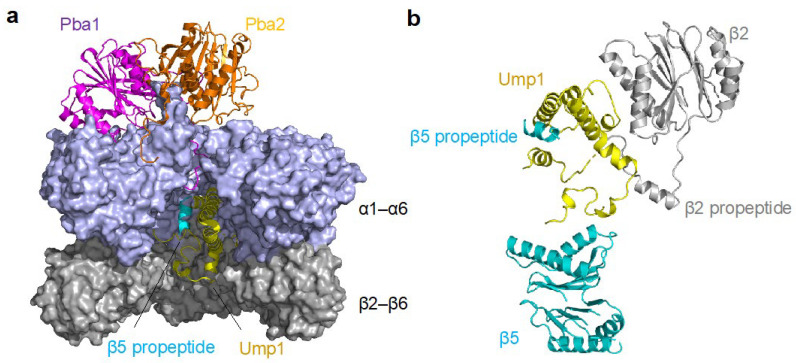
Structure of the pre-15S intermediate complex. (**a**) Cryo-EM structure of the pre-15S CP intermediate complex. The N-terminus of Pba1 extends to the CP interior and interacts with Ump1 and the propeptide of β5. α7 is omitted from the original structure (PDB 7LS6) for clarity. (**b**) Interaction between β2, β5, and Ump1. Ump1 makes contacts with both the propeptides and main bodies of β2 and β5. PyMOL was used for visualization (https://pymol.org/2/support.html), accessed on 27 April 2022.

Recent structural analysis revealed that the high affinity of Pba1–Pba2 for immature CPs is likely due to the association of Pba1 with another proteasome assembly chaperone, ubiquitin-mediated proteolysis 1 (Ump1, the mammalian ortholog is UMP1, also known as the proteasome maturation protein (POMP) and proteassemblin) (described below), and the β5 propeptide during CP assembly [26]. In the pre-15S complex, which consists of the α-ring, β2−6, Ump1, and Pba1–Pba2, the CP pore is open in a state distinct from that of the previously known open state, and the N-terminus of Pba1 extends through the pore into the CP interior, where Pba1 interacts with Ump1 and the β5 propeptide (Figure 3). Therefore, the absence of the propeptide of β5 and Ump1 in the mature CP likely weakens the binding between Pba1–Pba2 and mature CPs. The N-terminus of Pba1 also makes contact with the N-termini of all α subunits, and this likely underlies the mechanism by which Pba1–Pba2 and Pba3–Pba4 (described below) contribute to the correct arrangement of the α subunits. The N-terminus of Pba1 is thought to additionally assist in the discrimination between mature and immature CPs; Pba1–Pba2 likely fails to activate mature CPs upon binding to mature CPs due to the occlusion of the pore by the N-terminus of Pba1. In fact, a mutant Pba1–Pba2, lacking the N-terminus of Pba1, is able to activate mature CPs [26].

Further understanding of the interaction between Pba1–Pba2 and immature proteasomes was clarified by the cryo-EM structure of the Pba1–Pba2 complex with the pre-holoproteasome (immature, full CP with Ump1 and Pba1–Pba2 bound). On pre-holoproteasomes, the Pba1–Pba2 complex moves away from the central cavity of the α-ring to the α5 side, compared to when Pba1–Pba2 is in the 15S intermediate complex. Furthermore, Pba1 remains bound to the α5−α6 interface, while the binding between Pba2 and α7 is lost [27].

More recently, proteasome biogenesis-associated chaperone 5 (PBAC5), which forms a heterotrimer with the PAC1–PAC2 complex, was discovered in Arabidopsis [28]. PBAC5 contains an HbYX motif and binds between α4 and α5 in Arabidopsis. Although the function of PBAC5 has not yet been determined, homologous *PBAC5* genes also exist in fungi and metazoans, suggesting that it too plays an important role in CP assembly. Further work is required to elucidate what that role may be.

In comparison to the role PAC1–PAC2 plays in the proper assembly of the complete α-ring, PAC3–PAC4 is thought to play a more important role in the initial stages of α-ring formation. Knockdown experiments of each of the α-subunits revealed that the assembly of the α-ring likely starts with the formation of an intermediate formed of α4−7 as no intermediate was detected when any one of the α4−7 subunits was knocked down [25]. This core α4−7 intermediate was inhibited by PAC3 knockdown, indicating that PAC3–PAC4 is essential for the formation of this initial core intermediate. Knockdown experiments on α1, α2, and α3 further revealed that α1 and α3 can be incorporated into the α4−7 core intermediate independent of the other two subunits, whereas α2 cannot be incorporated without α1.

Both PAC3–PAC4 and Pba3–Pba4 bind most strongly to α5 in vitro, and co-crystallographic analysis of Pba3–Pba4 with α5 suggests that PAC3–PAC4/Pba3–Pba4 interact with α4, α5, and α6 on the β-ring side of the α-ring [19,29]. Furthermore, while α5 and α2 bind regardless of Pba3–Pba4, the binding of α5 and α4 is Pba3–Pba4 dependent, indicating that Pba3–Pba4 acts as a molecular matchmaker in forming the core α4−7 intermediate [30]. Co-crystallographic analysis also indicates that Pba3–Pba4 binds in a position that sterically clashes with β4. Thus, Pba3–Pba4 must dissociate from the CP intermediate before β4 binds to the α-ring. The release of PAC3–PAC4 from the CP intermediate is thought to also be coupled with the incorporation of β3 [19,31].

The lack of Pba3–Pba4 results in either the accumulation of dead-end complexes that do not incorporate the α4 subunit and instead appear to contain two copies of α2, or the induction of α4−α4 proteasomes with α-rings containing two copies of α4 instead of α3 [15,19,30]. The formation of the α4−α4 proteasome is also enhanced in mammals by the knockdown of PAC3 [32]. The α4−α4 proteasome can be induced by the reduction of *PAC3* mRNA expression caused by cadmium treatment and overexpression of the tyrosine kinases ABL and ARG, both of which inhibit α4 degradation. Therefore, the α4−α4 proteasome is thought to act in response to environmental stresses, as cells with enhanced α4−α4 proteasome formation are resistant to CdCl_2_ and CuCl_2_ [15,32].

### 2.2. Association of the β Subunits onto the Assembled α-Ring in an UMP1 Chaperone and Propeptide-Dependent Manner

The fully assembled α-ring acts as a scaffold for the assembly of the β-ring. Construction of the β-ring is aided by the chaperone molecule, UMP1 [33,34,35,36], as well as the N-terminal propeptide and the C-termini sequences of the β subunits themselves. The N-termini of the catalytic subunits β1, β2, and β5 and the non-catalytically active subunits β6 and β7 are translated with an additional propeptide sequence that is cleaved upon proteasome completion [37,38,39]. These propeptides assist with β-ring formation, inhibit proteolytic activity of the catalytic subunits before CP completion, and prevent N-acetylation of the β1 and β2 N-terminal catalytic Thr [40]. However, the propeptides of β1, β6, and β7 are not essential for proteasome maturation [31].

The order in which the β subunits are incorporated onto the α-ring was elucidated by knockdown experiments of each β subunit in animal cells [31]. First, β2 is incorporated onto the α-ring, followed by β3, β4, β5, β6, and β1, in that order, to complete the half-proteasome (-β7), into which β7 is lastly incorporated. In human cells, UMP1 is required for the binding of β2 to the α-ring, and the knockdown of UMP1 results in the accumulation of α-rings with no β subunits bound [31]. As Ump1 makes contact with both the propeptide and the main body of β2, it likely plays a major role in the positioning of β2. Upon its incorporation, the propeptide and C-terminus of β2 assist with the subsequent incorporation of β3, although neither are required for the incorporation of β2 itself. The long, C-terminus of β2 wraps around the β3 subunit within the same β-ring, and it was recently discovered that the propeptide of β2 also interacts with β3 and runs across the inner surface of the β-ring along the entire width of β3. Furthermore, the β2 propeptide is positioned between two segments of Ump1, β3, and β4, suggesting that the β2 propeptide may additionally facilitate the incorporation of β4 along with β3 [26,41]. The specific mechanisms governing the incorporation of the β5, β6, and β1 subunits remain to be fully resolved.

### 2.3. Association of Two Half-Proteasomes and Cleavage of the β Subunit Propeptides Completes the Assembly of the cCP

The final step in the assembly of the 20S proteasome is the association of two half-proteasomes, in a mirrored orientation. When β7 is incorporated into the half-proteasome (-β7), the C-terminal region of β7 inserts itself between the β1 and β2 subunits of the other half-proteasome, thus inducing the association of the two half-proteasomes [17,31,41,42]. The expression of a β5Δpro mutation, which lacks the N-terminal propeptide of β5, inhibits the association of the half-proteasomes, indicating that the β5 propeptide also plays a role in half-proteasome association. The overexpression of β7 suppresses the lethality caused by the β5Δpro mutation, suggesting that the propeptide of β5 is redundantly involved in the association of the half-proteasome with the C-terminus of β7 [17]. However, intriguingly, structural analysis of the pre-15S proteasome was unable to demonstrate the β5 propeptide projecting out of the half-proteasomes in such a manner as to facilitate dimerization, as was predicted. Instead, the ten most N-terminal residues of the β5 propeptide were in contact with Ump1 and α7 [26]. Thus, at present, structural analysis does not fully explain the mechanism by which the β5 propeptide aids the association of the half-proteasomes and the genetic interaction between β7 and the β5 propeptide.

In addition to the β5 propeptide and β7, Ump1 also facilitates the association of the half-proteasomes; the deletion of Ump1 and the β6 propeptide suppresses lethality caused by the β5Δpro mutant [17]. As such, both Ump1 and the β6 propeptide are assumed to inhibit the dimerization of the half-proteasomes, serving as a checkpoint to ensure that all β-subunits are bound before dimerization occurs. However, it was recently shown that the N-terminal region of Ump1 interacts with β7 in a manner dependent on the β7 propeptide. The phenotype in strains that express an N-terminal truncated variant of Ump1 is suppressed by overexpression of β7 [43]. Contrary to the previous checkpoint model for Ump1, this report suggests that Ump1 promotes the dimerization of the half-proteasomes through its interaction with β7.

Upon the proper dimerization of two half-proteasomes, the propeptides of the catalytic subunits β1, β2, and β5 are cleaved, exposing the catalytically active threonine residues at their N-termini. The β6 and β7 propeptides are also cleaved, and the PAC1–PAC2 complex and UMP1 bound to the half-proteasomes are degraded, thus completing CP maturation [13,33,34,44,45]. The 26S proteasome is finally formed by the association of the 20S CP with the 19S RP.

Prior to the assembly of the full 26S proteasome, the matured CP remains bound to the activator protein Blm10 (PA200 in human) until it associates with the RP [46,47]; Blm10 binds to the α-ring of the matured CP via its HbYX motif and forms a dome on top of the CP, thereby preventing proteins from entering the CP before the binding of the RP [46,48]. Blm10 also facilitates the nuclear import of the CP [49] and was shown to bind to CP intermediates [17,50,51]. Furthermore, the simultaneous deletion of Blm10 and the C-terminal truncation of β7 significantly retard growth with abnormal β2 processing and decreased proteasomal activity, although either mutation alone has little effect. Thus, Blm10 may also function to promote CP maturation [42]. Similarly, the simultaneous deletion of Blm10 and the expression of an RP *rpn2* mutant that is defective in the association of the RP with the CP causes abnormalities in the dimerization of the half-proteasomes and active site maturation, similar to those caused by the C-terminal defect in β7. In this regard, the 19S RP may have a functional commonality with Blm10 to assist CP maturation [42].

## 3. Assembly of the Immunoproteasome

The assembly of the immunoproteasome diverges from that of the cCP only in the formation of the β-ring, as the α-ring is identical to that of the cCP (Figure 2) [2]. The immunoproteasome is expressed in immune cells but is also upregulated in other cells upon stimulation by IFN-γ during inflammatory reactions. Due to its involvement in immune responses, the immunoproteasome must rapidly assemble by incorporating all the immunosubunits, β1i, β2i, and β5i, even in the presence of the constitutive proteasome subunits β1, β2, and β5.

### 3.1. Regulation of Immunoproteasome Expression by STAT1 and IFN-γ

The expression level of the immunoproteasome is regulated by its specific immunosubunits. The genes *PSMB9*, *PSMB10*, and *PSMB8* encode the catalytic β1i/LMP2, β2i/MECL-1, and β5i/LMP7 immunosubunits, respectively, and are upregulated by IFN-γ stimulation. Moreover, *PSMB9* and *PSMB8* are located in the MHC class II region in close proximity to the *TAP1* and *TAP2* genes [52]. In cells with a steady expression of the immunoproteasome, such as immune cells, STAT1 is involved in the constitutive expression of β1i and β2i, independent of IFN-γ [53]. On the other hand, in normal cells, transient expression of the immunosubunit genes is induced by IFN-γ. Interestingly, UMP1 expression is also stimulated by IFN-γ [33,36]; as UMP1 is degraded each time the proteasome is formed, an increase in UMP1 expression prevents the delay in proteasome formation caused by UMP1 depletion.

### 3.2. Assembly of the Immunoproteasome β-Ring Diverges from That of the Constitutive Proteasome with Respect to the Order of β Subunit Incorporation

**Figure 4:**
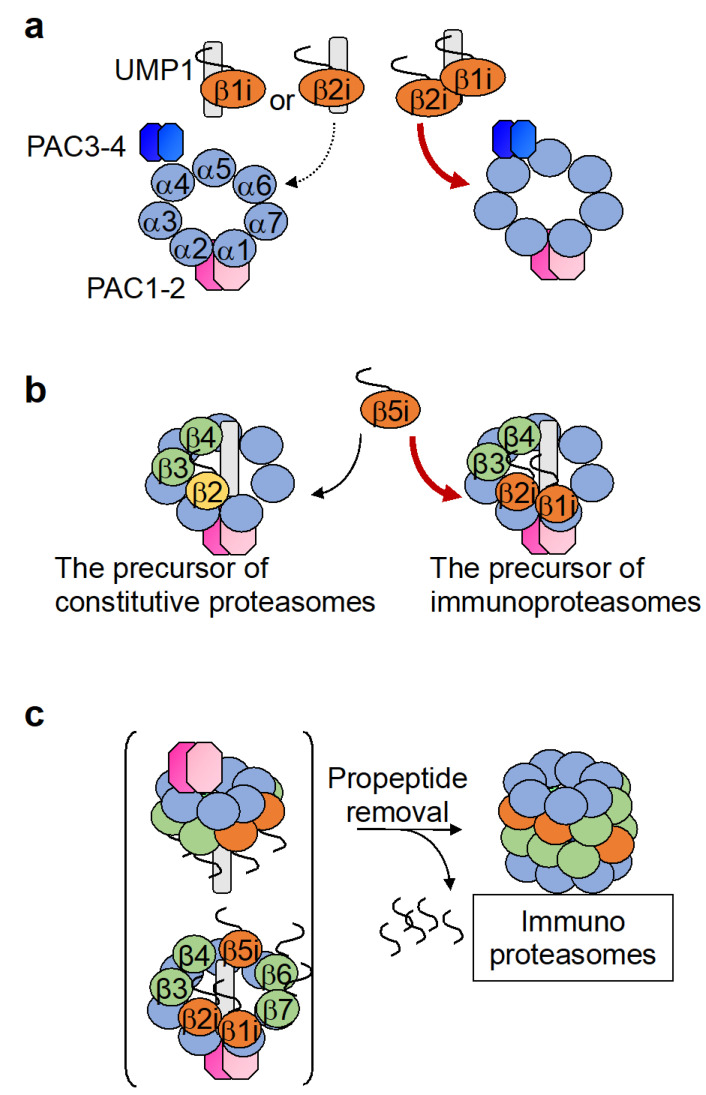
Incorporation of the three immunosubunits into the immunoproteasome. (**a**) β1i and β2i are mutually required for their incorporation during immunoproteasome assembly. Please note that the intermediate containing sole β1i or β2i with the α-ring has not been observed. (**b**) The precursor complex containing β1i and β2i promotes the incorporation of β5i. (**c**) The incorporation of β5i is necessary for removal of the β1i and β2i propeptides.

While the constitutive β subunits are assembled on the α-ring in the order of β2, β3, β4, β5, β6, β1, and β7 during the assembly of the cCP, knockdown experiments on the immunosubunits simultaneously with the constitutive catalytic β subunits using INF-γ-treated HeLa cells showed that both the knockdown of β1i or β2i causes an accumulation of α-rings with no β subunit attached [54]. Furthermore, an intermediate that contains the α-ring, β1i, and β2i can be detected even upon knockdown of β3. These results suggest that β1i and β2i are incorporated onto the α-ring first, in a simultaneous manner, during immunoproteasome formation (Figure 2 and Figure 4a). This model is consistent with a previous report in which intermediates containing β1i, β2i, β3, and β4 were observed [55]. However, although β1i and β2i promote each other’s incorporation during immunoproteasome formation, the degree of dependence of β1i and β2i on one another appears to differ [56,57,58]. Whereas intermediates containing the β2i precursor accumulate in concanavalin A (ConA) blasts from *β1i^−/−^* mice, the formation of proteasomes containing β1i, β2, and β5i was not inhibited in ConA blasts from *β2i^−/−^* mice, indicating that β2i is more dependent on β1i for its binding to the α-ring than β1i is on β2i [56]. Proteasomes with a mixture of constitutive and immune subunits, such as those that contain β1i, β2, and β5i, are called intermediate proteasomes and will be discussed further below.

Although β1i is incorporated onto the α-ring earlier than β1, the reason for this remains unknown. The propeptide of β1i is dispensable for its incorporation, although the efficiency of β1i incorporation decreases to about 70% in the absence of the propeptide [59]. Meanwhile, the propeptide of β2i contributes to the homogeneity of the immunoproteasome. When the propeptide of β2i is replaced with that of β2, this chimera β2i can incorporate into CPs that contain β1 and β5, and the incorporation of β5 into CPs containing β1i and β2i is also facilitated [56].

The formation of the immunoproteasome further diverges from that of the cCP with respect to the order of the β subunit incorporation in that β5i can be incorporated into the β-ring intermediate independent of β4. In contrast, constitutive β5 requires β4 for incorporation into the β-ring (Figure 2); the knockdown of β4 results in the identification of intermediates containing the α-ring and β1i, β2i, β3, and β5i, but none containing constitutive β5 [54]. Either β4 or β5i can be incorporated after β3 during immunoproteasome formation. Intermediates containing the α-ring, β1i, β2i, β3, and β4 were detected when β5i was knocked down. A β4-independent addition of β5i into the β-ring was also observed in cells expressing only constitutive β1 and β2 with the knockdown of β1i and β2i, indicating that the earlier incorporation of β5i is not dependent on β1i or β2i, but is a property of β5i itself. Interestingly, a β4-independent addition of β5i was not observed with chimeric proteins in which the propeptide of β5 was substituted with that of β5i, suggesting that the main peptide sequence, but not the propeptide, of β5i is important for β5i to be incorporated earlier than β4.

Meanwhile, unlike the β5 propeptide, which is not required for its own incorporation, the β5i propeptide, especially the N-terminal half, is required for β5i to be efficiently integrated into the immunoproteasome (Figure 4b) [60,61]. Constitutive β5 with the propeptide of β5i becomes efficiently incorporated into the immunoproteasome, while β5i with the propeptide of constitutive β5 is less efficiently incorporated into the immunoproteasome. This suggests that differences in the β5 and β5i propeptide sequences make constitutive β5 less likely to be added into immunoproteasome intermediates than β5i. As a result, the formation of CPs incorporating β1i, β2i, and β5 are less likely to occur [56]. Furthermore, the incorporation of β5i is required for the cleavage of the N-terminal propeptides of β1i and β2i, and this molecular mechanism also prevents the formation of CPs with a mixture of β1i, β2i, and constitutive β5 (Figure 4c) [57].

Besides being required for the incorporation of β1i and β2i onto the α-ring, as in the assembly of the cCP, UMP1 also serves to accelerate immunoproteasome biogenesis. Although both β5 and β5i interact with UMP1, β5i has a stronger binding affinity for UMP1 than β5 due to its propeptide sequence [62]. Together with the induction of UMP1 expression by INF-γ, these mechanisms result in the formation of immunoproteasomes four times faster than that of constitutive proteasomes [62]. However, the immunoproteasome is more unstable than the constitutive proteasome, with a half-life of 27 versus 133 h, respectively. The rapid turnover of the immunoproteasome is beneficial for the rapid adjustment of the immune system.

### 3.3. The Intermediate Proteasome Can Be Composed of a Mixture of Immuno- and Constitutive Subunits and May Play a Unique Role in Eliciting Immune Responses

Despite the mechanisms described above, which allow the immunoproteasome to efficiently incorporate three immunosubunits into a single immunoproteasome, intermediate proteasomes, which contain a mixture of constitutive and immunosubunits, are highly expressed in certain tissues [63,64]. The type I (β5i) intermediate proteasome, which incorporates β1, β2, and β5i, and the type II (β1i, β5i) intermediate proteasome, which incorporates β1i, β2, and β5i, are expressed in vivo. Type I intermediate proteasomes occupy from 0 to 50% of the total proteasome pool in vivo depending on the tissue type [65,66]. Specifically, type I intermediate proteasomes are highly expressed in liver, kidney, small intestine, colon, muscle, and dendritic cells [66,67,68], as well as in acute promyelocytic leukemia NB4 and histiocytic lymphoma U937 cell lines [65]. Meanwhile, type II intermediate proteasomes have been found to be primarily expressed in monocytes in vivo and account for 54% of their total proteasome pool [66]. Other cell lines, such as acute myelogenous leukemia KG1a cells, also express type II intermediate proteasomes [65].

β1i, β2i, and β5-incorporated intermediate proteasomes have been identified in INF-γ-treated β5i-deficient embryonic fibroblasts, β5-overexpressing cells, and the spleen of β5i-deficient mice. However, it remains unclear to what extent they exist in wild-type animals and cells. The presence of intermediate proteasomes incorporating β1i, β2, and β5 has been further reported in the liver of β5i-deficient mice. The existence of these intermediate proteasomes indicates that the β5 propeptide, although less efficient than β5i, does not preclude the simultaneous incorporation of β5 with β1i and β2i, and that once added, β5 has the potential to cleave the propeptides of β1i and β2i [69].

The formation of intermediate proteasomes is further believed to be dependent on β5i as β5i can be integrated into the forming CP without the incorporation of other immunosubunits and is required for the efficient cleavage of the β1i and β2i precursor sequences [54,57]. Indeed, β5i or β5 with a propeptide of β5i is incorporated into the cCP in cells that do not express β1i and the propeptide of β5i does not inhibit the incorporation of β5i into the cCP [61].

It is thought that intermediate proteasomes may contribute to the immune response by producing specific MHC class I binding peptides that are distinct from those generated by constitutive proteasomes and immunoproteasomes, thereby increasing the diversity of antigen peptides. Type I intermediate proteasomes specifically produce the MHC class I binding peptides MAGE (Melanoma Antigen Gene) A3_271–279_, and type II intermediate proteasomes generate MAGE A10_254–262_ and MAGE-C2_191–200_ [66,70,71].

Interestingly, the association of different types of half-proteasomes can form so-called asymmetric proteasomes, although they appear to only exist transiently in the middle of a change in intracellular proteasome types, such as when the expression of immunoproteasomes and intermediate proteasomes is increased immediately after IFN-γ stimulation. As a result, these asymmetric proteasomes include either both β1i and β1, or both β5i and β5 in a single molecule [72,73].

## 4. Assembly of the Thymoproteasome

The thymoproteasome is specifically, and likely stably, expressed in cTECs within the thymus [6]. The α-ring of the thymoproteasome is identical to that of the cCP and the immunoproteasome [2], while the β-ring formation of the thymoproteasome resembles that of the immunoproteasome. The catalytic subunit of the thymoproteasome is composed of β1i, β2i, and β5t; that is, the β5i subunit of the immunoproteasome is simply replaced by β5t. Collectively, β1i and β2i are incorporated first upon the formation of the α-ring, followed by β3, β5t or β4, β6, and lastly, β7 [54].

However, the mechanism by which β5t is incorporated independently of β4 differs from that of β5i. Unlike β5 chimeras with the propeptide of β5i, β5 chimeras with the propeptide of β5t become incorporated independent of β4. This indicates that the β5t propeptide is sufficient for β5t to be integrated into the β-ring earlier than β4 [54]. Furthermore, the processing of the β5t propeptide is more dependent on INF-γ than the processing of the β5i propeptide; β5t may be less readily incorporated into CPs with β1 and β2 than β5i is, and/or the β5t propeptide may be less easily processed by β1 and β2 [54].

Several mysteries regarding the specific expression of the thymoproteasome have yet to be fully resolved. Unlike that for the immune subunit β5i, the gene encoding β5t, *PSMB11*, lies adjacent to that for β5 and is not located in the MHC region. The transcription factor FOXN1 is directly involved in the expression of β5t, although FOXN1 is also expressed in mTECs that do not express β5t at detectable levels. Thus, the mechanism for induction of β5t expression specifically in cTECs remains elusive [74]. Additionally, β5i is also expressed in cTECs, although more than 90% of proteasomes in cTECs are thymoproteasomes [6]. While β5t is more readily incorporated into proteasome intermediates than β5i in cTECs, the mechanism for this preference remains unclear.

## 5. Techniques for the Identification and Analysis of β-Subunits

The constitutive, immuno-, and thymoproteasome β-subunits can be identified using subunit-specific antibodies to perform immunoblot analysis on lysates obtained from cells and tissues. β-subunits incorporated into CPs can be differentiated from free subunits by purification of CPs using anti-CP or anti-α-subunit antibodies followed by immunoblot analysis. It is also possible to detect all CP subunits via the two-dimensional polyacrylamide gel electrophoresis (2D-PAGE) of purified CPs with Coomassie staining, followed by tandem mass spectrometry analysis [6]. In addition to immunoprecipitation with anti-CP or anti-α-subunit antibodies, CPs can also be purified by column chromatography using chymotrypsin-like activity, which is the most important peptidase activity for proteasome-dependent protein degradation, as an indicator [75]. Specific peptidase activities can be measured using fluorogenic peptide substrates, such as Z-LLE-MCA for β1 caspase-like activity, Boc-LRR-MCA for β2 trypsin-like activity, and Suc-LLVY-MCA for β5 chymotrypsin-like activity.

## 6. Proteasome Assembly and Disease

It has become increasingly clear from the association between mutations in the immunosubunits and diseases that the assembly of the immunoproteasome is important for the maintenance of normal immune function (Table 1). Most notably, mutations in β5i manifest in autoimmune diseases, which have collectively been termed proteasome-associated autoinflammatory syndrome (PRAAS) [76,77,78]. PRAAS is commonly associated with symptoms such as joint contractures, muscle atrophy, microcytic anemia, and chronic atypical neutrophilic dermatosis with lipodystrophy and elevated temperature (CANDLE).

In β5i, mutations to G197V, G201V, T75M, and C135X were reported to cause PRAAS [79,80,81,82]. Among these, the accumulation of intermediates including unprocessed β1i and β2i was observed in G197V and G201V mutants, indicating that abnormal proteasome formation occurred [80,81]. T75 in β5i is also a highly conserved residue, similar to G197 and G201, and the T75M mutation disrupts the tertiary structure of β5i and reduces chymotrypsin-like activity. However, it remains unknown whether this mutation directly leads to defects in proteasome assembly [79]. Lastly, the C135X mutation deletes the C-terminal 141 amino acids of β5i. In silico analysis suggests that this mutation too causes improper formation of the immunoproteasome [82].

Currently, several mutations in the proteasome subunits, in addition to those in β5i, have been shown to lead to PRAAS. The heterozygous G156D β1i mutation reported by Kanazawa et al. causes pulmonary hypertension and immunodeficiency, in addition to PRAAS-like symptoms [83]. The G156 residue in β1 and β1i is highly conserved among multiple species and is located on the interface between the two β-rings in the assembled CP. Mutation of G156D may therefore inhibit the dimerization of the half-proteasomes. In line with this, the mutation of G156D results in impaired CP assembly, defects in the β1i incorporation, and detection of immature β1i. In addition to the G156D β1i mutation, a homozygous F14S mutation in β2i was recently reported to cause PRAAS [84]. This mutation resides in the β2i propeptide region and inhibits β2i incorporation. As a result, trypsin-like activity was decreased in mutant cell extracts. Mutations to the α7 and β7 subunits of the cCP are also thought to lead to PRAAS [85].

**Table 1:** Pathogenic mutations in the proteasome subunits and proteasome assembly chaperones.

Proteasome Subunits/ Proteasome Chaperone	Pathogenic Mutation	Reference
β1i	G156D	[83]
β2i	F14S	[84]
β5i	T75M	[79]
	C135X	[82]
	G197V	[80,81]
	G201V	
PAC2	Y223Sfs*2 & N225K	[86]
UMP1	truncation	[87]

Lastly, mutations in the proteasome chaperones, UMP1 and PAC2, have been reported [86,87]. These mutations are thought to inhibit the assembly of the proteasome, resulting in decreased protein hydrolysis activity. The accumulation of oxidized proteins, ubiquitinated proteins, and IFN-inducible proteins, which are typically degraded rapidly by proteasomes, leads to prolonged inflammation. The frameshift and N225K double mutation in PAC2 cause reduced proteasome expression and activity [86]. In UMP1, two mutations have been found, both of which result in truncated UMP1 proteins. Truncated UMP1 variants impair both the constitutive proteasome and immunoproteasome assembly [87]. The accumulation of ubiquitinated proteins and IFN-inducible proteins has been confirmed in PRAAS patients with such mutations in UMP1.

## 7. Conclusions

A variety of immune diseases have been reported to be the direct result of defects in the immunosubunits. The rapid and accurate assembly of the immuno- and thymoproteasomes in response to stimuli is critical for the correct function of these specialized proteasomes and the maintenance of normal immune function. Though extensive research has revealed much about the molecular mechanisms governing immuno- and thymoproteasome assembly, further research is required to clarify several points, such as the early incorporation of β1i, the organ-specific formation of intermediate proteasomes, and the preferential assembly of the thymoproteasome over the immunoproteasome in cTECs. Further elucidation of the molecular mechanisms of immuno- and thymoproteasome assembly may help to further our understanding of immune responses and to elucidate the causes of diseases, which manifest as a result of immuno- and thymoproteasome dysfunction.

## Data Availability

Not applicable.
